# Enhanced Biodiesel Production with Eversa Transform 2.0 Lipase on Magnetic Nanoparticles

**DOI:** 10.1021/acs.langmuir.4c02542

**Published:** 2024-11-26

**Authors:** Kaiany Moreira dos Santos, Juliana de França Serpa, Viviane de Castro Bizerra, Rafael Leandro Fernandes Melo, Paulo Gonçalves de Sousa Junior, Valdilane Santos Alexandre, Aluísio Marques da Fonseca, Pierre Basílio Almeida Fechine, Diego Lomonaco, José Cleiton Sousa dos Santos, Maria Cristiane Martins de Souza

**Affiliations:** † Instituto de Engenharia e Desenvolvimento Sustentável - IEDS, Campus das Auroras, Universidade da Integração Internacional da Lusofonia Afro-Brasileira - UNILAB, Rua José Franco de Oliveira, s/n - Zona Rural, Redenção 62790-970, CE, Brazil; ‡ Departamento de Engenharia Metalúrgica e de Materiais, Universidade Federal do Ceará−UFC, Campus do Pici, Bloco 729, Fortaleza CEP 60440-554, CE, Brazil; § Departamento de Química Analítica e Físico-Química, Universidade Federal do Ceará - UFC, Campus do Pici, Bloco 940, Av. Humberto Monte, 2825, CEP 60455760 Fortaleza, CE, Brazil; ∥ Laboratório de Produtos e Tecnologia em Processos (LPT), Universidade Federal do Ceará−UFC, Fortaleza 60440-900, CE, Brasil

## Abstract

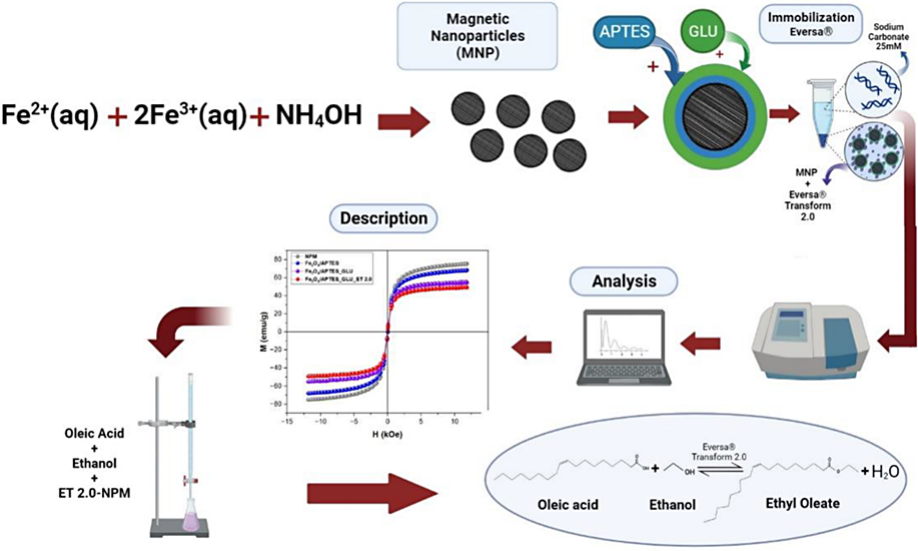

This research investigated the usefulness of magnetic iron oxide nanoparticles (Fe_3_O_4_) as a support to immobilize the lipase Eversa Transform 2.0 (ET 2.0) to obtain an active and stable biocatalyst, easily recoverable from the reaction medium for applications in the production of biodiesel. Biodiesel was an alternative fuel composed mainly of fatty acid esters with strong transesterification and esterification capabilities. The study focused on the esterification of oleic acid with ethanol to synthesize ethyl oleate. Magnetic nanoparticles were prepared by coprecipitation, then activated with glutaraldehyde and functionalized with γ-aminopropyltriethoxysilane (APTES). The optimal conditions for immobilizing ET 2.0 were pH 10, 25 mM sodium carbonate buffer, an enzymatic load of 200 U/g, and 1 h of contact time, obtaining 78% yield and enzymatic activity of 205.9 U/g. Postimmobilization evaluation showed that the immobilized enzyme performed better than its free form. Kinetic studies were conducted under these optimized conditions (2–96 h at 150 rpm and 37 °C). The biocatalyst was tested for the synthesis of ethyl oleate using oleic acid as the substrate and ethanol, achieving a conversion of 88.1%. Subsequent recirculation tests maintained approximately 80% conversion until the fourth cycle, confirming the sustainability of ester production. Molecular docking studies revealed that the binding affinity for the enzyme-docked oil composition was estimated at −5.8 kcal/mol, suggesting that the combination of the substrate and lipase was stable and suitable for esterification.

## Introduction

Beginning in April 2023, Brazil has reached a pivotal point in its sustainable energy policy by introducing a 12% (B12) biodiesel blend in diesel fuels.^1^ This move is part of a broader, proactive government mandate to increase this blend to 13% (B13) by April 2024, underscoring Brazil’s unwavering commitment to adopting greener energy alternatives as part of its national agenda.^2^ While Brazil has historically been at the forefront of renewable energy consumption, a surprising 6.5% decline in biodiesel consumption last year has raised urgent questions.^3^ These questions concern not only the sustainability of biodiesel as an alternative but also the overall effectiveness of Brazil’s renewable energy initiatives. This changing landscape requires a comprehensive reassessment of Brazil’s existing renewable energy strategies and the scientific technologies that support them.^4^

Biodiesel, a renewable biofuel composed primarily of fatty acid esters, has emerged as a compelling alternative to conventional fossil-based diesel.^5^ The production of biodiesel is based on two primary chemical processes: transesterification and esterification.^6,7^ These processes involve the catalytic reaction of vegetable oils or animal fats with primary alcohols to produce biodiesel and glycerin.^8,9^ Of the primary alcohols, methanol is preferred over ethanol in industrial applications. This preference is mainly due to the cost-effectiveness of methanol and its inherently higher reactivity, which often results in higher biodiesel yields.^10,11^

In parallel, the global chemical industry is undergoing a transformative phase characterized by an accelerating shift toward greener, more sustainable processes.^12,13^ Within this transformative framework, enzymes have gained prominence as eco-efficient catalysts, driven by their exceptional specificity and reduced byproduct formation.^14,15^ This high specificity offers two advantages: it increases the overall yield and eliminates the need for complex postreaction separation processes.^16,17^ Both aspects contribute significantly to biodiesel production’s economic viability and environmental sustainability.^18,19^

Enzymatic catalysis offers several advantages over traditional chemical catalysis, particularly biodiesel production.^20^ These advantages include milder reaction conditions, higher substrate specificity, and significantly lower energy consumption.^21,22^ In addition, the adaptability of enzymatic processes allows for using a wide range of feedstocks, including low-quality oils and fats that are typically unsuitable for chemical catalysis, thereby expanding the range of potential sources for biodiesel production.^23−26^ Of the many commercially available lipases, Eversa Transform 2.0 (ET 2.0) stands out as a promising catalyst for biodiesel production.^27,28^ ET 2.0, introduced by Novozymes, is a liquid lipase derived from genetically modified *Thermomyces lanuginosus*.^29,30^ It offers several advantages, including high substrate specificity, robust catalytic activity under moderate conditions, and extended shelf life. Together, these attributes position ET 2.0 as a desirable candidate for large-scale industrial applications.^23−25,31^

Despite their compelling advantages, enzymes in their native, soluble state face several operational challenges, including thermal instability and complex recovery processes from reaction mixtures.^32,33^ These issues limit their operational longevity and reusability.^34,35^ Enzymes can be immobilized onto solid supports.^36−38^ This immobilization increases thermal and operational stability and facilitates recovery and reuse in multiple reaction cycles.^39−42^ Among various types of solid supports, magnetite (Fe_3_O_4_) nanoparticles have gained attention as a particularly effective medium for enzyme immobilization.^43−45^ These nanoparticles are characterized by low toxicity, a large surface area conducive to enzyme attachment, and superparamagnetic properties allowing easy separation from reaction mixtures.^46,47^

Consequently, magnetic nanoparticles (MNPs) are a viable medium for enzyme immobilization because of their unique physicochemical properties.^48−50^ In particular, their superparamagnetic properties allow for easy postreaction recovery and reuse in subsequent catalytic cycles.^51,52^

The overarching goal of the current investigation is multifaceted. First, it aims to synthesize and rigorously characterize magnetite (Fe_3_O_4_) nanoparticles using a streamlined, efficient method. Second, the study aims to evaluate the suitability of these synthesized Fe_3_O_4_ nanoparticles as a solid support for the immobilization of ET 2.0. Various operating parameters will be investigated to achieve an optimized immobilization process, including pH conditions, enzyme loading rates, and duration of enzyme–substrate contact time. In addition, the study addresses the intrinsic properties of the magnetite material and its operational stability, all aimed at improving its applicability in the synthesis of ethyl oleate.

The production of ethyl oleate is of great industrial importance because of its wide range of applications in various sectors. A fatty acid ester derived from oleic acid and ethanol, ethyl oleate is widely used as a green solvent, emulsifier, and plasticizer in the polymer industry. In addition, its biocompatibility and low toxicity make ethyl oleate an ideal candidate for pharmaceutical applications, particularly as a solubilizer in drug formulations and as a carrier in controlled drug delivery systems. Ethyl oleate has also attracted attention in the food industry as a flavouring agent and biodiesel production as a renewable fuel. Its multifunctionality and renewable nature make ethyl oleate, a material of considerable interest for sustainable industrial applications.^53−55^

## Methods

### Materials and Methods

Novozyme Latin America Ltd.a supplied ET 2.0. It is located in Araucária, Paraná, Brazil. Gamma-aminopropyltriethoxysilane (APTES), a 25% (w/v) solution of grade II purity glutaraldehyde, p-nitrophenyl butyrate (*p-*NPB), and p-nitrophenol (*p*-NP) were purchased from Sigma-Aldrich, São Paulo, Brazil. All other analytical grade reagents were purchased from Distribuidora Cequímica, Fortaleza, state of Ceará, Brazil.

### Synthesis of Magnetic Nanoparticles (NPM)

Magnetite nanoparticles (Fe_3_O_4_) were prepared by using a modified coprecipitation technique.^56^ Briefly, ferrous sulfate heptahydrate (FeSO_4_-7H_2_O; 1.55g) and ferric chloride hexahydrate (FeCl_3_-6H_2_O; 3.00g) were dissolved quantitatively in deionized water and combined in a glass beaker. The pH of the solution was carefully adjusted to 3.0 using a 5% (v/v) hydrochloric acid solution. The mixture was then thermally stabilized at 80 °C and magnetically stirred at 1,200 rpm for 30 min. After that, 30 mL of ammonium hydroxide (NH_4_OH, analytical grade) was gradually added and stirring continued for another 30 min, culminating in the precipitation of magnetite nanoparticles. The chemical reaction is shown below:
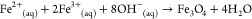


The resulting residue was washed with distilled water to a neutral pH, followed by a single ethanol wash to remove residual water molecules.^19^ The precipitate was magnetically separated and then dried in a desiccator. The synthesized material was named NPM.^19^

### Treatment with Gamma-Aminopropyltriethoxysilane (APTES)

Magnetic nanoparticles were dispersed in 200 mL of 95% ethanol and subjected to ultrasonication for 60 min. After ultrasonication, 10 mL of APTES was added, and the system was further ultrasonicated at 60 °C for another hour. The resulting material was magnetically separated and dried in a desiccator according to a modified protocol from a previous study.^57^

### Activation of Support with Glutaraldehyde (GLU)

NPMs were incubated in a 25% (w/v) glutaraldehyde solution as described by a previous study.^58^ An aliquot of 25 μL glutaraldehyde was mixed with 0.01 g magnetite nanoparticles, and the system was stirred at 25 °C for 2 h. Excess glutaraldehyde was then removed by washing three times with 5 mmol L^–1^ sodium phosphate buffer (pH 7.0). Glutaraldehyde-treated nanoparticles were designated NPM-GLU.

### Immobilization of Enzymes

One ml of sodium phosphate buffer (5 mmol L-1, pH 7.0), and 3 μL of the enzyme were added to a 0.01 g sample of magnetite nanoparticles (Fe_3_O_4_). The admixture was maintained at 25 °C and subjected to mechanical stirring at 45 rpm for 1 h. The enzyme-support contact time was studied at 1, 2, 5, and 8 h, activity measurements of the immobilized enzyme were performed, and hydrolytic activities were determined to allow the calculation of immobilization parameters. The immobilized enzyme was then magnetically separated from the aqueous medium.

### Measurement of Enzyme Activity

The catalytic efficiency of both free and immobilized forms of ET 2.0 was assessed by using p-nitrophenyl butyrate (*p-*NPB) as a substrate. Assays were performed at a pH of 7.0 and a temperature of 25 °C, following a modified methodology in a previous study.^59^

### Determination of Enzyme Activity

Enzyme activity was quantified spectrophotometrically using a 50 mmol L^–1^ solution of p-nitrophenyl butyrate (*p-*NPB) in isopropanol. A reaction mixture was prepared by combining 50 μL of *p-*NPB, 2.5 mL of 25 mmol L^–1^ sodium phosphate buffer (pH 7.0), and either 50 μL of enzyme sample or 10 mg of immobilized biocatalyst. The reaction was incubated at 25 °C, and the p-nitrophenol formed was quantified spectrophotometrically at 348 nm. One unit of enzymatic activity was defined as the amount of enzyme hydrolyzing 1 μmol of *p*NPB per minute under assay conditions.^58^

### Comprehensive Characterization of Supports and Biocatalysts

Extensive characterization of both the support materials and the biocatalysts was performed using advanced analytical techniques to understand their properties, functionalities, and potential limitations thoroughly.

### Vibrating Sample Magnetometry (VSM)

VSM analysis was performed at a constant temperature of 300 K to generate magnetic hysteresis curves. These curves provide critical data on the magnetic behavior and stability of the nanoparticles under different magnetic field strengths. The VSM instrument was calibrated with standard reference materials before the measurements were taken to ensure the accuracy of the magnetic moments obtained. For each magnetic field applied, the resulting magnetic moments were carefully normalized to the mass of nanoparticles, allowing a more accurate comparison and interpretation of the magnetic properties of different samples.^60^

### Scanning Electron Microscopy (MEV) and X-ray Fluorescence (XRF)

The supports and biocatalysts’ morphological and elemental composition were investigated using scanning electron microscopy (SEM) imaging and XRF spectroscopy. A QUANTA 450 FEG microscope was used for SEM analysis. The samples were prepared by attaching them to carbon tape and then sputter-coating them with a thin layer of silver using Quorum QT150ES metallization equipment. This process enhanced the conductivity of the samples and allowed high-resolution imaging under a 20 kV electron beam. In parallel, XRF analysis was performed using a SHIMADZU model EDX-7000 instrument equipped with a rhodium tube, applying a 4 kV power setting to the powdered samples. The synergy of SEM and XRF provides morphological details and elemental composition, thus offering a comprehensive characterization.^60^

### Fourier Transform Infrared Spectroscopy (FTIR)

FTIR spectroscopy was used to identify the functional groups and detect possible interactions between the supports and the enzymes. Spectra were obtained using a PerkinElmer 2000 spectrophotometer, covering a wavenumber range from 4000 to 400 cm^–1^. Before spectral acquisition, samples were predried to reduce interference from water absorption. Samples were then dispersed in potassium bromide (KBr) at a ratio of 1:10, and the mixture was compressed into translucent tablets using a hydraulic press. This preparation method improved the quality of the FTIR spectra, allowing for a more reliable interpretation of the functional groups and binding interactions present.^60^

### Effect of pH on Enzymatic Activity

The impact of pH on catalytic activity was studied using buffers of different pH values and *p*NPB as substrate. Buffers were sodium acetate (pH 5.0), sodium phosphate (pH 6.0–8.0), and sodium carbonate (pH 9.0 and 10.0). The investigation occurred under the same conditions as the enzyme immobilization step mentioned above, with the buffer change for each pH analyzed.

### Effect of Enzyme Loading

Enzyme loading was evaluated from 50 to 300 U g^–1^. Immobilization was performed by combining 0.01g of magnetic nanoparticles with 1 mL of sodium phosphate buffer (pH 7.0) and adjusting the amount of enzyme according to the desired loading. Postimmobilization, enzyme activity was evaluated as described above in the section Determination of Enzyme Activity.

### Impact of Contact Time

The effect of contact time on immobilization efficiency was evaluated at 1-, 2-, 5-, and 8-h intervals, with all other conditions held constant. After each interval, the activity of the immobilized enzyme was evaluated.

### Synthesis of Ethyl Oleate

Esterification reactions were performed in 2.0 mL microtubes containing 0.6 g oleic acid, 151 μL ethanol (maintaining a 1:1 molar ratio), and 0.01 g immobilized biocatalyst. The reaction was conducted at 37 °C with orbital shaking at 150 rpm. The acidity index was determined according to AOCS method 5–40.^61^

### Operational Stability and Kinetics

#### Kinetic Investigation of Esterification Reaction

Kinetic studies were meticulously performed over an extended time ranging from 2 to 96 h to determine the optimal reaction time for converting oleic acid to ethyl oleate. For these experiments, inert plastic microtubes were used to contain the reaction mixture, which consisted of 0.01 g of magnetite-based nanoparticles, 8 μL of enzyme solution, 0.6 g of oleic acid, and 151 μL of ethanol. The reaction mixture was subjected to controlled agitation at 150 rpm for the predetermined intervals.

After the reaction, 0.5 g of the resulting ester product was carefully transferred to an Erlenmeyer flask. To this was added 25 mL of neutralized commercial alcohol and two drops of phenolphthalein as an indicator. Titration was then performed with a 0.1 M potassium hydroxide (KOH) solution. The volume of KOH consumed during the titration was meticulously recorded, allowing accurate calculation of the conversion of oleic acid to ethyl oleate.

#### Evaluation of Operational Stability in Esterification

The operational robustness of the biocatalyst in the esterification process was studied using successive cycles of ethyl oleate synthesis. In each cycle, 0.01 g of the enzymatic derivative ET 2.0-NPM was used. Before initiating each reaction cycle, the enzymatic derivative was magnetically separated and subjected to a thorough wash with hexane to remove residual reaction products and unreacted substrates. Subsequent esterification cycles were performed using the methods described in section Synthesis of Ethyl Oleate.

### Characterization of Esters

#### Gas Chromatography/Mass Spectrometry (GC/MS)

To determine the ester content (C) of the produced biodiesel samples, GC/MS analysis was performed according to the method described in EN 14103 with modifications. This European standard can be used to measure the contents of linolenic acid and fatty acid methyl esters (FAMEs) in biodiesel by gas chromatography (GC). This approach allows the calculation of the proportion of FAME in biodiesel and ensures that the fuel meets quality requirements. Approximately 50 mg of the biodiesel sample was placed in a 2 mL vial containing 1 mL of methyl nonadecanoate solution (10 mg/mL). This mixture was then injected (1 μL) into a SHIMADZU QP-2010 ULTRA gas chromatograph–mass spectrometer equipped with a (5%-phenyl)-methylpolysiloxane (DB-5) capillary column (30 m × 0.25 mm × 0.25 μm film thickness) using helium as the carrier gas in splitless mode.^62^

#### Nuclear Magnetic Resonance (NMR)

One-dimensional spectra of hydrogen nuclear magnetic resonance (^1^H NMR) and carbon nuclear magnetic resonance (^13^C NMR) were obtained by using a Bruker spectrometer, model Advance DRX-500, at the Northeastern Center for Application and Use of Nuclear Magnetic Resonance, Federal University of Ceará (CENAUREMN-UFC). The experiment was performed at a hydrogen frequency of 500 MHz to verify the chemical shifts and multiplicities of isotopes in the carbon chains of formed esters. The solvent used to dissolve the samples was deuterated chloroform (CDCl_3_). Analyses were performed in 5 mm tubes.^63^

#### High-Performance Liquid Chromatography (HPLC)

An aliquot of the reaction mixture at the optimal conversion point was analyzed using high-performance liquid chromatography (HPLC). The system was equipped with a Shimadzu LC-18 column and an ultraviolet detector. Analysis was performed using an isocratic method with a solvent composition of 60% methanol and 40% acetonitrile. Injection volume was 20 μL at a flow rate of 1 μL per minute for 5 min at 40 °C. The wavelength selected for analysis was 254 nm, where the spectrum showed higher absorption levels.^64^

#### Homology Modeling

First, comparative modeling of the ET 2.0 was performed in four stages.

#### Identification and Selection of Protein-Fold

The amino acid sequence of ET 2.0, with CAS number 9001–62–1 from Sigma-Aldrich, was submitted for comparative analysis using the essential local alignment search tool (BLAST) software^65^ and Protein Data Bank (PDB) archive. A protein related to the amino acid sequence, the lipase enzyme classified as hydrolase, was identified from *Aspergillus oryzae*, expressed using *Escherichia coli*-*Pichia pastoris* shuttle. The target protein was obtained from PDB (https://www.rcsb.org/) under code 5XK2.

#### Alignment of Target and Mold Sequences

The alignment between the sequences was conducted by using MODELER software.^66^

#### Model Construction and Optimization

The model’s construction was also performed using Modeler 10.4 software,^66^ resulting in a new protein named Eversa. Eversa was evaluated according to objective function and stereochemical parameters.^67^

#### Protein Validation

Model validation was performed at stereochemical, conformational, and energetic levels. The generated model’s quality was validated usingachandran chart^68^ with the PROCHECK software, which evaluated its three-dimensional structure and indicated the possible stereochemical quality.^69^

#### Protein Preparation

Protein generated by Eversa homology was subjected to charge correction and hydrogen atom addition process using the AutoDock Tools software.^70^

#### Obtaining the Ligand

The structure of oleic acid (Figure S1) was created using ChemDraw 3D software and then minimized using an MM2 force field with an RMS gradient of 0.0001.^71^ The configuration was performed for structural optimization ingadro software,^72^ using the Merck molecular force field (MMFF94) with 500 interaction cycles and the steepest descent algorithm with a convergence limit of 10 × 10^–7^.^73^ The optimized structure was then converted to the pdbqt format.

#### Molecular Docking and Visualization of Calculations

Molecular docking simulations were performed using AutoDock Vina software 74, which considered rigid proteins and flexible ligands. A grid configuration was set for both calculations with parameters specific to the enzyme’s active sit.^74,75^ Energy profiles of ligand–receptor interactions were also evaluated, and the anchored positions were visualized using BIOVIA Discovery Studio software.^76^

## Discussion and Results

### The Role of Glutaraldehyde in Enzyme Immobilization

Our results showed glutaraldehyde activation resulted in a superior immobilization yield of 92% and derivative activity of 195.36 U g^–1^, compared to an 83.1% yield and 101.25 U g^–^^1^ derivative activity without activation. This underscores the critical role of glutaraldehyde in enzyme immobilization, a conclusion consistent with Modenez et al., who observed that optimized concentrations of glutaraldehyde significantly affected the yield of soybean oil transesterification.^77^ Our results are also consistent with Brandão Júnior et al.,^62^ who used glutaraldehyde in a similar immobilization procedure and reported conversion yields ranging from 68.7 to 82.2% for the esterification of babassu oil.^62^

### Influence of Nanoparticle Concentration and Steric Hindrance

Modenez et al.^77^ pointed out that higher concentrations of nanoparticles resulted in higher lipase loading but never reached 100%, possibly due to inaccuracies in the Bradford method of quantification.^77^ In our study, the improved yield and activity with glutaraldehyde activation may also be due to a more effective enzyme-nanoparticle interaction. This could be further investigated regarding steric hindrance and improved quantification methods.

### Chemical Modifications and Enzyme Conformation

Both Modenez et al. and Miranda et al. addressed the consequences of different concentrations of glutaraldehyde, explicitly mentioning that excessive amounts could lead to intraenzyme cross-linking and potentially alter the enzyme’s three-dimensional structure.^77,78^ This provides a plausible explanation for the significant increase in derivative activity observed in our glutaraldehyde-activated sample. This chemical modification likely induced a favorable conformational change or increased the rigidity of the enzyme, thereby enhancing its activity.

### Functional Group Density and Immobilization Efficiency

Research indicates that APTES is efficient in forming surfaces with amino groups, enabling a solid connection with enzymes. These amino groups, particularly at varying pHs, can interact with the enzyme through electrostatic attraction and hydrophobic interactions, contributing to stable immobilization. The connection between nanoparticles and enzymes occurs at the interface between solid and liquid, favoring the effectiveness and stability of the process.^79^ Glutaraldehyde acts as a cross-linking catalyst, establishing covalent bonds between enzymes and functional supports. This stabilizes the enzyme structure and increases its activity and stability compared to immobilization techniques without cross-linking. These covalent connections prevent the enzyme’s deactivation through continuous use.^80,81^ Gao et al. emphasized the need for a carefully regulated degree of oxidation of the dextran polymer to ensure an optimal density of functional groups.^82^ Although our study did not directly measure the thickness of functional groups, the superior derivative activity in the glutaraldehyde-activated sample may imply an optimized functional group interaction, consistent with the literature.^31,83^ In our study, the immobilization parameters of Fe_3_O_4_/APTES_ET 2.0 and Fe_3_O_4_/APTES_Glu_ET 2.0 were investigated (Table 1), focusing on the influence of glutaraldehyde. The immobilization yield for samples with and without no glutaraldehyde was approximately 82.04 and 90.5%, respectively.

**Table 1:** Comparative Analysis of Immobilization Efficacy for Fe_3_O_4/_APTES_ET 2.0 and Fe_3_O_4_/APTES_Glu_ET 2.0: Detailed Assessment of Immobilization Yield (IY), Theoretical and Actual Derivative Activity (AtT and AtD), and Activity Recovery Post-Immobilization (AtR)

biocatalyst	yield (%)	theoretical activity (U g^–^^1^)	derivative activity (U g^–^^1^)	activity recovered (U g^–^^1^)
Fe_3_O_4_/APTES_ET 2.0	83.1 ± 1.3	166.3 ± 1.5	101.25 ± 1.2	60 ± 0.1
Fe_3_O_4_/APTES_Glu_ET 2.0	92 ± 1.1	184 ± 0.5	195.36 ± 0.2	95 ± 0.08

A significant increase in derivative activity was observed in the presence of glutaraldehyde (196.17 U/mg) compared to its absence (101.25 U/mg). This contrasts the findings of Martínez-Sanchez et al., who reported specific activities of TLL and ET against p-nitrophenyl butyrate (pNPB) of about 185 and 350 U/mg for free lipases, respectively. Their study also highlighted the phenomenon of hyperactivation upon immobilization, with TLL activity increasing 2.5-fold at lower enzyme loadings. However, the ET exhibited less hyperactivation, resulting in similar final activities for both immobilized biocatalysts.^84,85^ The difference in immobilization results between our study and that of Martínez-Sanchez et al. could be attributed to differences in the supports used (Fe_3_O_4_/APTES and octyl agarose) and the inherent properties of the lipases involved. The variation in enzyme loadings and the nature of the immobilization mechanism (the presence of glutaraldehyde in our case) could further contribute to these differences. Our results highlight the critical role of support modification and enzyme source in determining biocatalysts’ immobilization efficiency and activity profiles.

### Characterization of Magnetic Nanoparticles and Biocatalysts

#### Investigation of Magnetic Loading Influence on Fabricated Supports

**Figure 1:**
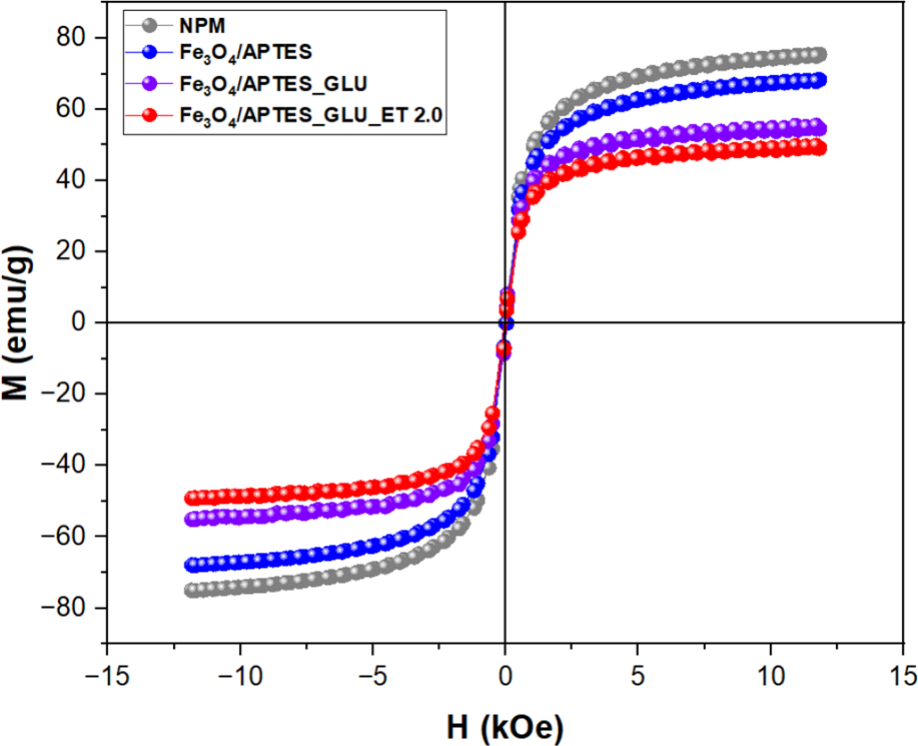
Magnetic characterization of synthesized materials and biocatalysts. NM: magnetic nanoparticles (magnetite [Fe_3_O_4_]); Fe_3_O_4_/APTES: magnetite functionalized with APTES (γ-aminopropyltriethoxysilane); Fe_3_O_4_/APTES_GLU: magnetite functionalized with APTES and activated with GLU (glutaraldehyde); Fe_3_O_4_/APTES_GLU_ET 2.0: EVERSA 2.0: enzyme immobilized on magnetite functionalized with APTES and activated with glutaraldehyde.

The detailed study of the impact of magnetic loading on the fabricated supports, with particular focus on the samples designated NPM, Fe_3_O_4_/APTES, Fe_3_O_4_/APTES_GLU, and Fe_3_O_4_/APTES_GLU_ET 2.0, provides critical insight into their magnetic properties. The observed saturation magnetization (M_s) values — approximately 75, 70, 55, and 50 emu/g for the respective samples (Figure 1) — reflect a discernible decrease upon functionalization of the naked magnetic particles (NPM) with APTES (γ-aminopropyltriethoxysilane) and glutaraldehyde. This trend is consistent with Costa et al.,^86^ who also reported a similar decrease in M_values upon APTES and glutaraldehyde coating of Fe_3_O_4_ nanoparticles.^86^ The subsequent immobilization of ET 2.0 further reduces the M_s values, supporting the idea that adding nonmagnetic entities adversely affects the magnetic properties. However, the resulting nanosystem retains sufficient magnetization for effective magnetic separation, as Califano et al.^85^ confirmed the successful functionalization and utility of Fe_3_O_4_/APTES_GLU_ET 2.0 as a biocatalyst support.^87^ The decrease in saturation magnetization (M_s) values upon modification is consistent with the study by Satpute et al.,^88^ who observed a reduction in M_s from 66.61 to 17.52 emu/g with increasing Y^3+^ substitution in cobalt ferrite nanoparticles (CoY_*x*_Fe_2–*x*_O_4_). This was attributed to the replacement of magnetic Fe^3+^ ions by nonmagnetic Y^3+^ ions, significantly affecting the superexchange interactions and, consequently, the magnetic properties. Factors such as porosity, homogeneity, morphology, and cation distribution at lattice sites, as discussed by Satpute, are critical in influencing these magnetic parameters. Despite the reduction in M_s due to APTES, glutaraldehyde and ET 2.0 functionalization, the magnetic response of the nanosystem remains sufficient for practical magnetic separation applications. This suggests that, similar to the findings by Satpute, external factors in the synthesis process critically determine the final magnetic properties of the nanocomposites.^88^

#### Integration of Scanning Electron Microscopy (SEM) and X-ray Fluorescence (XRF) for Material Characterization

**Figure 2:**
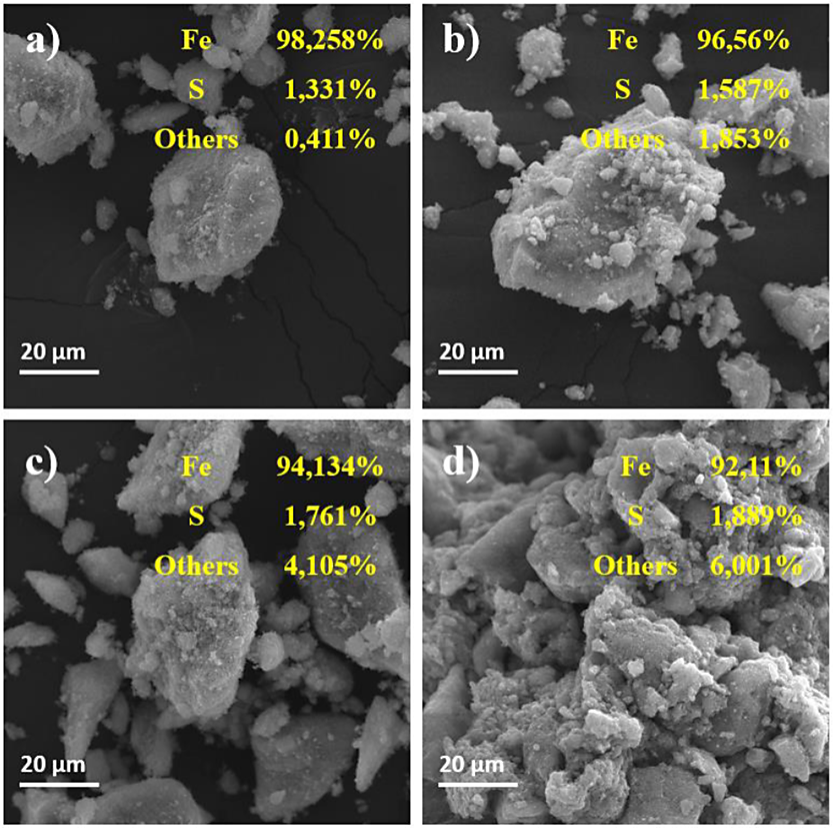
SEM and FRX images. NPM: (a) magnetic nanoparticles (magnetite [Fe_3_O_4_]); (b) Fe_3_O_4_/APTES - magnetite functionalized with APTES (γ-aminopropyltriethoxysilane); (c) Fe_3_O_4_/APTES_GLU - magnetite functionalized with APTES and activated with GLU (glutaraldehyde); (d) Fe_3_O_4_/APTES_GLU_ET 2.0 - enzyme EVERSA TRANSFORM 2.0 immobilized in magnetite functionalized in APTES and activated with glutaraldehyde.

SEM images and X-ray fluorescence (XRF) spectra of the nanomaterials—NPM, Fe_3_O_4_/APTES, Fe_3_O_4_/APTES_GLU, and Fe_3_O_4_/APTES_GLU_ET 2.0— are shown together in Figure 2 (panels a–d). SEM micrographs reveal nuanced differences in the morphological characteristics and porosity of the different engineered supports. A striking change in material porosity is indicative of rapid compositional changes. XRF spectral analysis supports minor elemental shifts, particularly a marginal decrease in iron (Fe) content associated with the appearance of sulfur (S) and other elements. This elemental redistribution supports the hypothesis that additional components are incorporated during the APTES and GLU functionalization processes. All investigated materials show a laminar surface topology. The fusion of SEM and XRF analytical techniques provides a robust validation of the successful immobilization of ET 2.0 onto the Fe_3_O_4_/APTES_GLU_ET 2.0 support, thereby confirming its utility as a biocatalytic platform.^89^ SEM images and XRF data show that the amount of other elements increases while the amount of iron decreases. This is particularly true in samples (c) and (d), where variation in elemental composition is most visible.^90,91^ The morphology of (c) shows smaller and potentially more porous particles, indicating a larger interaction surface and, therefore, more effective immobilization of other compounds. The morphology of (d) is more complex and irregular, which may indicate a more significant amount of immobilized material. The change in particle morphology in different samples also shows that the surface modification or coverage is different in degree.

#### Fourier Transform Infrared Spectroscopy (FTIR) for Molecular Characterization

**Figure 3:**
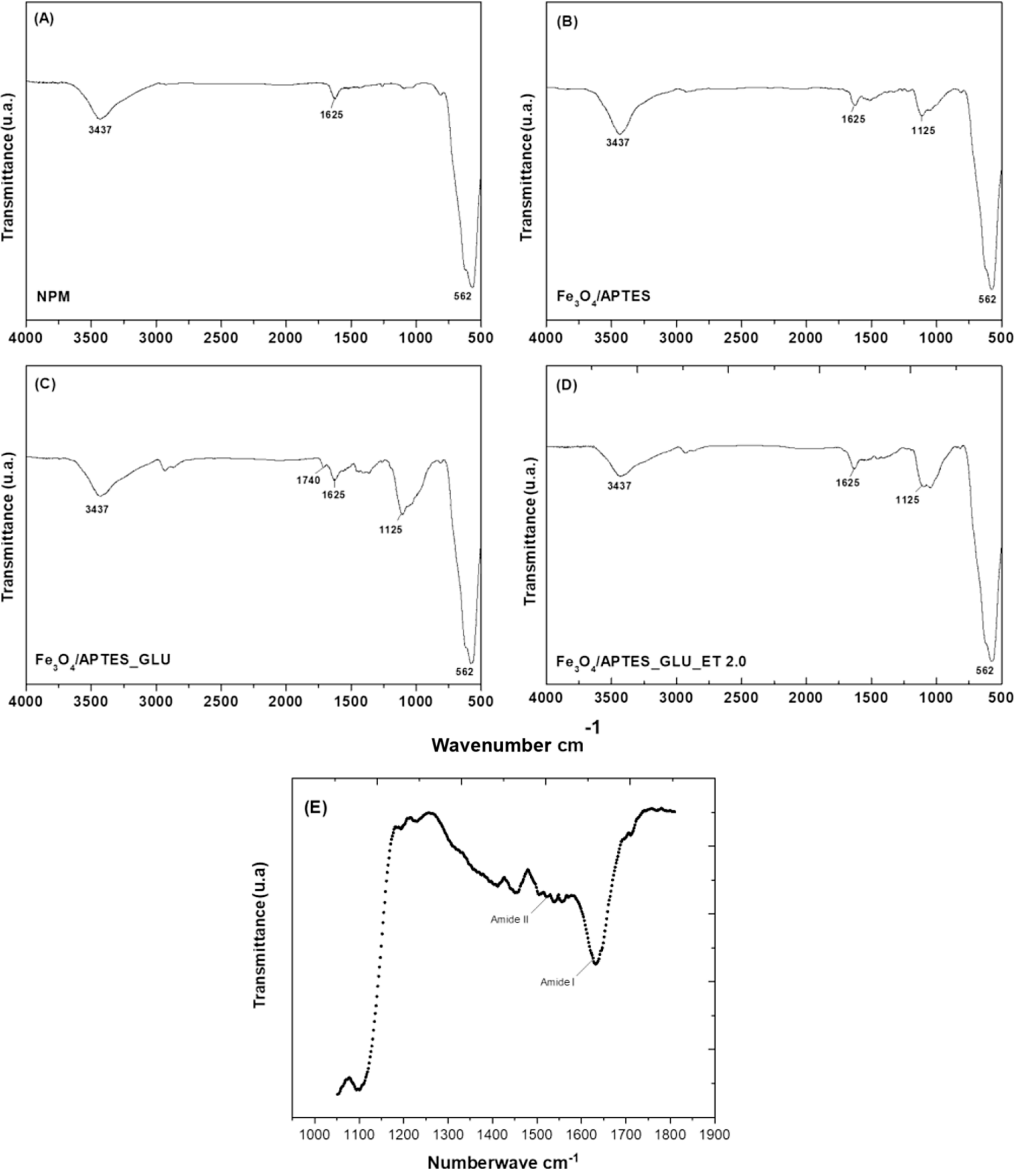
FTIR spectra of the samples. (A) Magnetic nanoparticles (magnetite [Fe_3_O_4_]); (B) Fe_3_O_4_/APTES - magnetite functionalized with APTES (*g*-aminopropyltriethoxysilane); (C) Fe_3_O_4/_APTES_GLU - magnetite functionalized with APTES and activated with GLU (glutaraldehyde); (D) Fe_3_O_4_/APTES_GLU_ET 2.0 - Eversa Transform 2.0 enzyme immobilized onto magnetite functionalized in APTES and activated with glutaraldehyde; (E) visualization of the Amide I and Amide II bands.

Fourier transform infrared spectroscopy (FTIR) can confirm the presence and integrity of functional groups introduced during the APTES and glutaraldehyde functionalization processes. FTIR can detect characteristic vibrational modes associated with enzyme functional groups, providing insight into the binding mechanisms and orientation of the immobilized enzyme.^92^Figure 3 shows the FTIR spectra for both the benchmark and synthesized nanomaterial systems. A ubiquitous absorption band observed at 562 cm^–1^ in all sample types confirms the presence of Fe–O vibrational modes, indicative of the spinel lattice in the magnetic phase.

In the spectrum shown in Figure 3, two prominent bands appear at 1625 and 3437 cm^–1^, which are attributed to the bending vibrations of adsorbed H_2_O molecules and the stretching vibrations of surface hydroxyl OH groups, respectively. The other material systems described in Figure 3 consistently observe these spectral features. Figure 3 shows the FTIR spectrum corresponding to the Fe_3_O_4_/APTES composite. An absorption band manifesting at 1125 cm^–1^ is attributed to Fe–Si bonds, confirming the hypothesis suggested by the diffraction pattern of the Fe_3_O_4_/APTES system. This spectral evidence confirms the successful surface modification of Fe_3_O_4_ nanoparticles by APTES functionalization. The FTIR spectrum shown in Figure 3 examines the molecular changes after glutaraldehyde activation of Fe_3_O_4_/APTES. While the characteristic magnetite bands remain unchanged, a new spectral peak appears at 1740 cm^–1^, corresponding to the stretching vibration of the carbonyl (C=O) group in the aldehyde molecule, confirming the activation of the support matrix with glutaraldehyde. Figure 3 shows that the band at 1,740 cm^–1^ decreases, probably due to the binding of the aldehyde group of glutaraldehyde to the amine of the enzyme added to the reaction medium. The amide I and amide II bands in the spectrum, located in the 1640–1560 cm-1 region, may indicate the presence of proteins. The amide I band, situated between 1650 and 1600 cm^–1^, is linked to the stretching vibration of the C=O group of peptide bonds.^93^ This can reveal the secondary structure of proteins, such as α-helices and sheets β. The amide II band, which appears between 1550 and 1500 cm^–1^, is associated with N–H bending and C–N stretching in peptide bonds.^94^ The appearance of these bands in the spectrum indicates the presence of the Eversa enzyme in the sample. The identification and interaction of the enzyme with other components, such as oleic acid, in the study in question, are reinforced by the bands that show protein structures by spectroscopy.^95,96^

#### Investigation of pH-Dependent Activity for Free and Immobilized Lipase Enzymes

**Figure 4:**
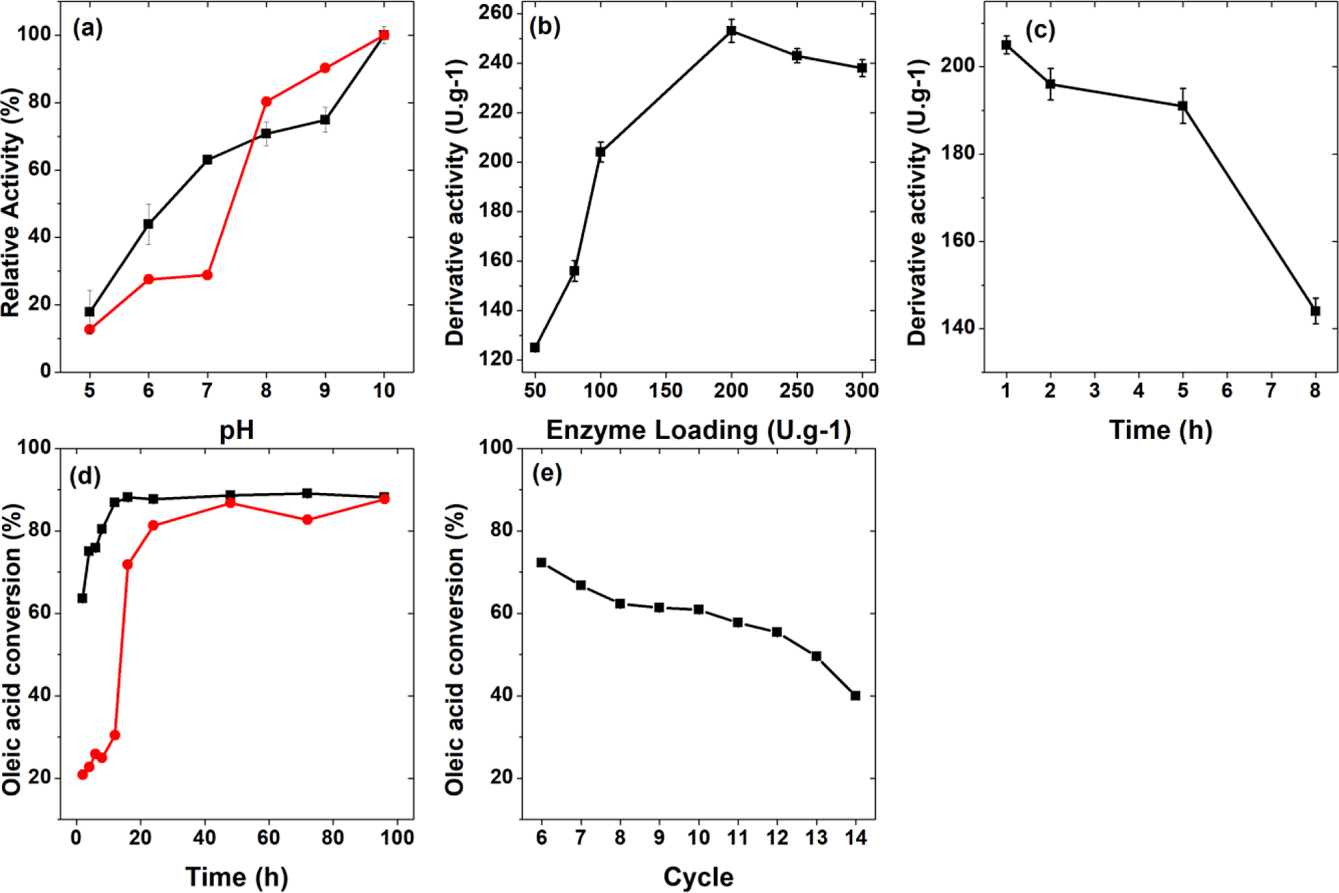
(A) Influence of pH on the activities of free ET 2.0 (red circle) and ET 2.0 immobilized onto magnetic nanoparticles (■). The activity was determined by using *p*NPB (enzyme loading: 80 U *p*NPB g^–1^), (B) effect of enzyme loading (Atd, U.g^–1^) on the immobilization of ET 2.0 onto magnetic nanoparticles, (C) effect of contact time (Atd, U g^–1^) on the immobilization of ET 2.0 onto magnetic nanoparticles, (D) synthesis of ethyl oleate using immobilized ET 2.0 NPM (■) and free ET 2.0 (red circle) with an enzyme load of 200 U g^–1^, at 37 °C and 150 rpm stirring for 2, 4, 6, 8, 12, 24, 48, 72, and 96 h, (E) operational stability of ethyl oleate synthesis using ET 2.0-NPM with an enzyme load of 200 U.g^–1^ at 37 °C. Each cycle lasted 16 h, with stirring at 150 rpm.

Figure 4 provides a compelling illustration of how pH can affect enzymatic performance, with free and immobilized enzymes showing optimal activity at an alkaline pH of 10. Enzymatic activity, measured in relative activity units for the free enzyme, increased from 0.6136 at pH 5 to 0.9402 at pH 10. When comparing, it is possible to observe that at pH 5 the activity of the immobilized enzyme was 57 times greater than that of the free enzyme, with a corresponding decrease in associated errors. This suggests improved enzyme performance and stability at higher pH levels. This is consistent with the documented preference of lipases for alkaline conditions, which is attributed to increased structural stability and the potential for conformational rearrangements that increase active site accessibility to substrates. The immobilized enzyme exhibited a more pronounced increase in activity from pH 5 to 10, with activity units rising from 35 to 151, also reflecting a reduction in error values. Notably, the immobilized enzyme exhibited higher activity under acidic conditions (pH 5 and 6) than its free counterpart. This improved performance can be attributed to the protective effects of immobilization, which can shield the enzyme’s active site and maintain its integrity against the denaturing effects of acidic pH. The enhanced stability and activity of the immobilized enzyme in acidic conditions could be because of several factors, including a change in the pH of the microenvironment around the enzyme, increased resistance to conformational changes, and the protective matrix provided by immobilization. These observations are consistent with previous studies by Arana-Peña et al. and Miranda et al., who also noted the stabilizing effects of immobilization on enzyme activity.^78,97^ Error analysis across the pH spectrum for free and immobilized enzymes provides insight into the variability and reliability of activity measurements. Lower error values at higher pH levels for both enzyme forms indicate more consistent activity, likely because of the increased stability of the enzyme structure under alkaline conditions. This research underscores the importance of pH in the performance of lipase enzymes and highlights the benefits of immobilization in improving enzyme stability over a range of pH conditions. The results have significant implications for applying these biocatalysts in industrial processes where pH can vary widely and stability under such conditions is paramount.

#### Effect of Enzymatic Load on the Activity of Immobilized Lipase

In a recent study investigating the impact of enzyme loading on the activity of immobilized lipase onto MNPs, an interesting trend was observed that underscores the nuanced balance between enzyme loading and activity. As the enzyme load increased from 50 to 200 U/g, there was a corresponding increase in activity, which peaked at 200 U/g. However, further increases to 250 and 300 U/g resulted in a decrease in activity. This pattern is consistent with established enzyme immobilization and stability principles, as discussed extensively in the literature. According to de Souza, the initial increase in activity with increasing enzyme concentration can be attributed to a higher probability of substrate-enzyme interactions because of an increased number of active sites.^19^ as noted by Silva et al., beyond a certain threshold, the benefits of additional enzymes are counteracted by diffusional limitations and steric hindrance, which is corroborated by our findings at higher enzyme loads.^98^ Immobilization capacity and activity recovery can explain activity reduction beyond optimal loading, which is critical for efficient immobilization.^99^ When enzyme molecules are crowded on the support surface, they are likely to interfere with each other’s access to substrates, resulting in reduced activity, a concept supported by Zhang et al.^100^ Immobilization of enzymes onto MNPs offers a compelling method to enhance enzyme properties such as stability and activity.^101^ The superparamagnetic nature of these supports facilitates easy recovery and reuse, which is critical for cost-effective industrial processes.^102^ In addition, MNPs provide increased enzyme loading capacity and reduced mass transfer resistance, thereby improving the overall efficiency of enzymatic reactions.^103^ The observed decrease in enzyme activity at higher loadings is consistent with studies showing that excessive enzyme concentrations can lead to protein–protein interactions that prevent the conformational flexibility necessary for optimal catalytic function.^104^ This highlights the importance of determining the optimal enzyme load for immobilization, balancing the increased activity with the potential for diffusional limitations and steric hindrance.^105^

#### Effect of Contact Time

The optimization of enzyme immobilization on MNPs is a multifaceted process that requires careful consideration of various parameters, including enzyme loading, contact time, and enzyme concentration. Our investigations show that both the amount of immobilized enzyme and the duration of immobilization significantly affect the enzymatic activity. When immobilization time and enzyme concentration were further investigated, we observed a peak activity at a 1-h optimal contact time, followed by a decrease in activity over time. This is consistent with the findings of Monteiro et al., who reported that increased immobilization time can lead to active site blockage due to overcoverage by excess enzyme.^106^ The effect of contact time on immobilized activity showed maximum activity was reached after 1-h incubation, with a subsequent decrease, indicating an optimal interaction window (1–5 h) for enzyme support. This behavior mirrors the interaction patterns seen in other studies by Costa et al. and Huang and Cheng, where longer contact times lead to decreased catalytic activity, possibly because of enzyme denaturation or the formation of obstructive multiple bonds between the enzyme and the support.^86,107^

Huang and Cheng reported that a short contact time is required for enzyme immobilization on nanoparticles because of the large available surface area, which reduces the mass transfer resistance. In addition, modification of the support with APTES and glutaraldehyde facilitated rapid binding of the enzyme through covalent interactions between the aldehyde group of glutaraldehyde and the amine groups of the enzyme.^86,107^ The interplay between immobilization variables and enzyme properties is critical. The results underscore the importance of a balanced approach to enzyme immobilization, where immobilization time, enzyme loading, and concentration are carefully calibrated to avoid detrimental effects on enzyme structure and activity. In addition, the stability of the enzyme under varying pH conditions and its operating environment must be considered to maintain its catalytic ability.^108,109^

#### Catalytic Esterification

The synthesis of ethyl oleate from oleic acid using enzymatic esterification represents a compelling process for biodiesel production. Figure 4 illustrates the catalytic efficiency of the ET 2.0-NPM biocatalyst under optimal immobilization conditions over an extended reaction time of 2–96 h at 37 °C with continuous stirring. The enzymatic kinetics, reflected by the conversion rates of oleic acid to ethyl oleate, show a high degree of conversion (88.1%) within the first 16 h, indicating a robust catalytic process.

This pattern of activity is consistent with the reported literature, where Souza (2013) found lipase from *Candida antarctica* B (CALB) maintained 85% activity over multiple cycles. Miranda et al. documented an initial high yield with a subsequent decline upon repeated use. Our results are consistent with Brandão Júnior et al., who observed variable conversion rates over multiple cycles without significant loss of catalytic potential.^19,62,78,110^ Several factors may contribute to the observed decrease in activity: (1) Enzyme stability: As the reaction time increases, the enzyme may undergo conformational changes, resulting in decreased strength and activity. This is consistent with the gradual decline in activity after the optimal 16-h reaction time; (2) Diffusion limitations: The initial high activity suggests minimal resistance to mass transfer. However, as the reaction progresses, diffusional limitations may become more pronounced, possibly because of product accumulation or changes in the enzyme microenvironment; (3) Enzyme desorption and aggregation: Over extended periods, desorption of the enzyme from the support or aggregation phenomena may occur, which can affect catalytic efficiency. The high conversion rate achieved early in the reaction underscores the efficiency of the ET 2.0-NPM biocatalyst. Compared to the literature, the faster conversion rate indicates a superior catalytic process, possibly due to reduced diffusion effects or more effective enzyme immobilization on the MNPs.

Enzyme immobilization reduces capital costs and is considered an investment in the production process, as it reduces reaction time and provides better enzyme substrate results.^111^ All support costs and the stability of the chosen enzymes are essential parameters for selecting the best immobilization method to be used.^49^ The application of immobilized enzymes as industrial catalysts has had its potential for years, since the 1960s, with proven production cost savings of up to 40% and labor reduction.^112^ The costs of the enzyme immobilization process may decrease based on advances in microbial biodiversity, which may lead to more frequent use of immobilized enzymes in biodiesel industries, as well as in food industries, since the magnetic nanoparticle support is nontoxic, performing an efficient and sustainable interaction with the enzymes, becoming an industrially attractive biocatalyst.^113,114^

In 2022, the study of^115^ concluded that enzymes applied in industries, the so-called industrial enzymes, have a global market that has been growing in the last two decades, with total market revenue currently of US$ 10.6 billion, identifying that improvements in the efficiency processes of these enzymes used industrially, in addition to being fundamental, are necessary for the expansion of more sustainable and promising processes.^116^ Several studies prove the high enzymatic activity capabilities of enzymes immobilized on adequate supports, especially magnetic nanoparticles, all with successful and superior results compared to free enzymes.^117,118^

Lipase enzymes immobilized on magnetic nanoparticle support catalyze various reactions for biodiesel production, such as transesterification and esterification reactions.^119,120^ Although these supports are economically attractive, some are still not viable due to catalytic performance and operational stability, which must be obtained from the immobilized enzyme.^121^ The high operational stability of a biocatalyst immobilized from magnetic nanoparticles, without the need for regeneration by organic solvent, also becomes economically viable, minimizing costs in industrial and production processes.^122,123^ In addition to promising applications in biotechnology industries, the catalytic activities of nanomaterials are high and flexible, similar to those of enzymes, as they have low cost and high operational stability.^124^ Magnetic nanoparticles are highly versatile, facilitating the process of separating enzymes during immobilization, which promotes reuse. This is one of the main benefits of using magnetic nanoparticles in enzyme immobilization. This magnetic recovery reduces considerable production costs for industries, together with immobilized enzymes, which present more excellent activity and pH and temperature stability when compared to free enzymes, which do not use supports.^125^ Magnetic nanoparticles are highlighted as excellent enzyme immobilization supports because they are easy to separate and recover during the reaction, in addition to having a large surface area and high mass transfer capacity, but specific optimization protocols are still needed to increase catalytic activity, as well as recyclability.^126^

The industrial production of enzymatic biocatalysts, especially the immobilization of lipases on magnetic nanoparticle supports, becomes favorable on a large scale because it facilitates the transition from laboratory scales to suitable industrial scale manufacturing configurations, being versatile methods of adaptation.^127^ Magnetic nanoparticles support reducing the use of hazardous and waste-generating reagents, offering sustainable syntheses. They are also prominent in the synthesis of fine chemicals, allowing the production of fragrances, natural products, and flavours, all of their uses being sustainable.^128^ Biotransformation becomes versatile, favoring the synthesis of compounds. In addition to being useful in synthesizing biomolecules, it contributes applications in biosensors, detecting condensation reaction products from analytical applications.^129^

Magnetic nanoparticles have a green chemistry approach and a high magnetic response capacity, which helps simplify steps during recovery and reduces time and costs compared to traditional separation techniques.^49,115^ They also provide a high surface area to volume ratio and various binding sites for enzyme immobilization.^120^ Magnetic nanoparticles offer a controlled microenvironment for enzyme immobilization, allowing for increased stability, preventing enzyme leaching, sustaining catalytic activity, and being biodegradable and biocompatible.^125^

#### Operational Stability

Operational stability is critical to enzyme-mediated reactions, particularly in industrial biodiesel production, where reusability and sustained catalytic performance are essential for economic viability. Figure 4 and associated data reflect the stability and efficiency of the ET 2.0-NPM biocatalyst over extended reaction cycles. The performance of the biocatalyst over 14 cycles of 16 h each shows a promising start, with a conversion of 88.1% for oleic acid to ethyl oleate in the first cycle. Stability is maintained until the fourth cycle, with conversions around 80%. However, a gradual decline is observed from the fifth cycle onward, dropping to 39.9% in the final cycle. This trend is similar to the findings of Brandão Júnior et al. and Remonatto et al., who reported a slight decrease in activity after several cycles, highlighting the ability of the enzymes to maintain performance over multiple reuses.^62,130^ The operational stability observed in this study is commendable compared to other research. Razack and Duraiarasan (2016) found sustained activity after 20 cycles, and Remonatto et al. observed only a 25% decrease after 3 cycles.

Martínez-Sanchez et al. and Bresolin et al. reported less robust results but demonstrated the potential for immobilized enzymes in biodiesel applications.^84,131−133^ Modifying MNPs with APTES and glutaraldehyde is advantageous for maintaining enzyme activity over successive cycles.^31,134^ The APTES modification protects against nanoparticle oxidation, while the glutaraldehyde provides functional groups for enzyme attachment. This dual modification strategy supports the assertion that the immobilization technique employed can significantly influence the operational stability of the biocatalyst. ET 2.0-NPM shows impressive efficiency in the production of ethyl oleate. The initial high conversion rates and relatively sustained performance over multiple cycles suggest that this biocatalyst is a strong candidate for replacing more harmful chemical catalysts. The ability to easily separate and recover the biocatalyst using a magnetic field adds to its industrial appeal. When magnetic nanoparticles immobilized in lipase enzymes are found in complex environments, their behavior can vary in environments with magnetic materials and components, directly influencing their performance.^135^ Nanoparticles can interact with each other and initiate aggregate formation, which can alter the magnetic properties of these particles.^136^ External magnetic fields can also affect magnetic nanoparticles, as they influence the alignment of the nanoparticles.^137^ It can also influence the interactions between enzymes and the support, directly influencing the catalytic efficiency.^127^ They can also benefit in environments with magnetic material even if they have different properties, which can complement the system, making it more efficient and versatile.^138^

Magnetic nanoparticles become magnetic when they are near an external magnet. When this magnet is removed, its magnetic state is undone, which means that the particles are not in an “active” state at all times.^139,140^ In other words, the magnetic field influences the position and direction of the magnetic nanoparticles in the reactions.^141^ The main structure of magnetic nanoparticles is their magnetic core, which is surrounded by an active group coating. The magnetic characteristics of the particles are due to the response factor to the external magnetic field applied externally.^142^ Aggregation, or accumulation, of magnetic nanoparticles, can be avoided by stabilization through electrostatic or steric repulsion.^143^ Other magnetic materials in the environment where magnetic nanoparticles support the enzymatic biocatalyst lead to various specific behaviors, which will directly depend on the magnetic interactions and the characteristics of the components involved.^144^

### Characterization Analysis

#### GC/MS

Table S1 shows the composition of the esters in the reaction medium and their proportions. The proportion of ethyl oleate is 80.3%, making it the predominant product among the components of the medium.^145^ This enrichment of the sample in the ester fraction demonstrates the efficiency of the ethyl ester production catalyzed by ET 2.0. The chemical profile of these components shows an ester composition of more than 89%, ensuring that the substrates present have been converted into their respective esters.^146^

#### ^1^H NMR

In the ^1^H NMR spectrum (500 MHz, CDCl_3_) of the reaction medium, it is possible to observe the signals of the hydrogens for the predominant ester present in the reaction system. Figure S2 highlights some significant chemical shifts for ethyl oleate. Expansions for the hydrogens highlighted in the structure have been included to identify and assign the signals to their respective chemical shift values and the corresponding diversity of isotopes. The signals near δ 5.34 were attributed to the hydrogens of the sp^2^ carbons present in the carbon chain of ethyl oleate. Because of the trans conformation present in this region of the molecule, signal splitting is observed, resulting in the formation of a multiplet. This chemical profile and splitting are characteristic of trans alkenes because of the couplings between the isotopic nuclei.^147^ In addition, there is a more excellent s-orbital character than methyl groups, resulting in these hydrogens experiencing greater deshielding because of the increased electronegativity of the double bond, leading to higher chemical shift values. The signal at δ 4.13 is a quartet, corresponding to the diversity and chemical shift attributed to the hydrogens directly bonded to the oxygen of the ester group. These hydrogens are directly bonded to an electronegative atom, which explains the deshielding observed by the shift and the quartet diversity corresponding to the neighboring methyl hydrogens.^148^ The triplet observed at δ 2.77 is characteristic of methylene hydrogens in the α position relative to the carbonyl of esters. The diversity of this signal corresponds to the neighboring environment of these hydrogens being a methylene group, −CH_2_. The signals observed near δ 1.29 and δ 1.31 are related to the common methylene groups in the rest of the ester carbon chain. Finally, the signal at δ 0.87 was attributed to the methyl group at the end near the ester carbonyl, as it has more excellent shielding, a lower chemical shift, and a corresponding diversity. Other signals in the spectrum are related to other esters in lesser proportions in the reaction medium.

#### HPLC

The chromatogram of the reaction medium Figure S3 was processed, and the peak with a retention factor equivalent to 7.791 was attributed to the methyl ester.^149^ Because of its high content, previously determined by GC/MS, ethyl oleate showed a higher absorption at a wavelength of 254 nm. This absorption is expected for structures with carbonyl functional groups, such as esters.^150^ The other products, such as the unconverted acids, have significantly different retention factors, showing a greater affinity for the polar solvent.^151^ Because of the low concentration of the sample and their absorption peaks outside the ideal range, the remaining molecules are not well described in the chromatogram.

#### Protein Modeling

**Figure 5:**
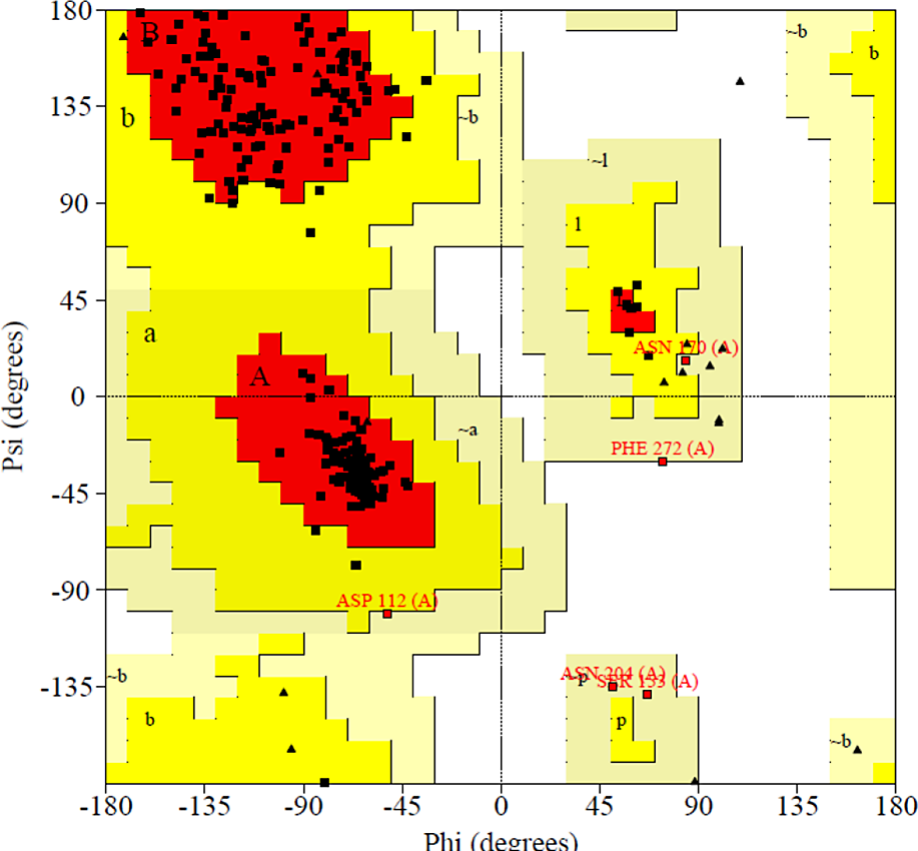
Ramachandran plot of the patterned Eversa.

Ramachandran plot (Figure 5) shows that 91.5% of the residues are in the favorable regions (red region). In addition, 6.5% of the residues are in the allowed regions (regions a, b, l, p, yellow), 1.6% are in the generously allowed regions (regions ∼a, ∼b, ∼l, ∼p, light yellow), and 0.4% are in the unfavorable regions (empty region). The residues found in the unfavorable regions reflect the structures used as templates, some located at the ends of the protein. Therefore, the Ramachandran plot data support the obtained model. This alignment recognized structurally conserved and variable regions, highlighting the structurally equivalent residues in the primary sequence in the lipase identification process.^152^ Studies found in the literature by Qin et al. the Ramachandran assessment indicated that 89.7% of waste was found in the most suitable areas and 99.7% in authorized areas, with only 0.3% in unauthorized areas. This indicated that the created model has sufficient accuracy for future studies.^153^ In another research conducted by Tütüncüo and collaborators, the Ramachandran graph was used to confirm the predicted structures of thermoalkaliphilic lipases. In this case, around 91.2–91.8% of the waste was found in the most favored areas, without outliers (waste that was not within the permitted areas), which confirms the excellence of the model.^154^

#### Interaction between Substrate and Lipase

Molecular docking studies were performed to validate the approaches used to explain the observed results for Eversa. According to the literature, van der Waals strengths and hydrogen bonds were favorable, with binding affinities indicated by molecular coupling studies.^155^ Therefore, for immobilization, the Eversa lipase was structurally studied by molecular modeling, with an overview of lipase binding using AutoDock Vina and DS software to predict its affinity, orientation, and environmental surfaces.^156^ The Eversa catalytic site is a triad represented by Ser 153, His 268, and Asp 206,^74,75,157^ with the serine residue acting as a nucleophile in the carbonyl group of the substrate for esterification bioreactions within the substrate pocket.^158,159^ Substrates of suitable molecular forms can occupy these subsites and undergo catalysis, such as the carboxylic acids in the tucuman and cupuaçu oil composition. The binding affinity for the composition oil anchored with the enzyme was estimated at −5.8 kcal/mol (Table S2).

**Figure 6:**
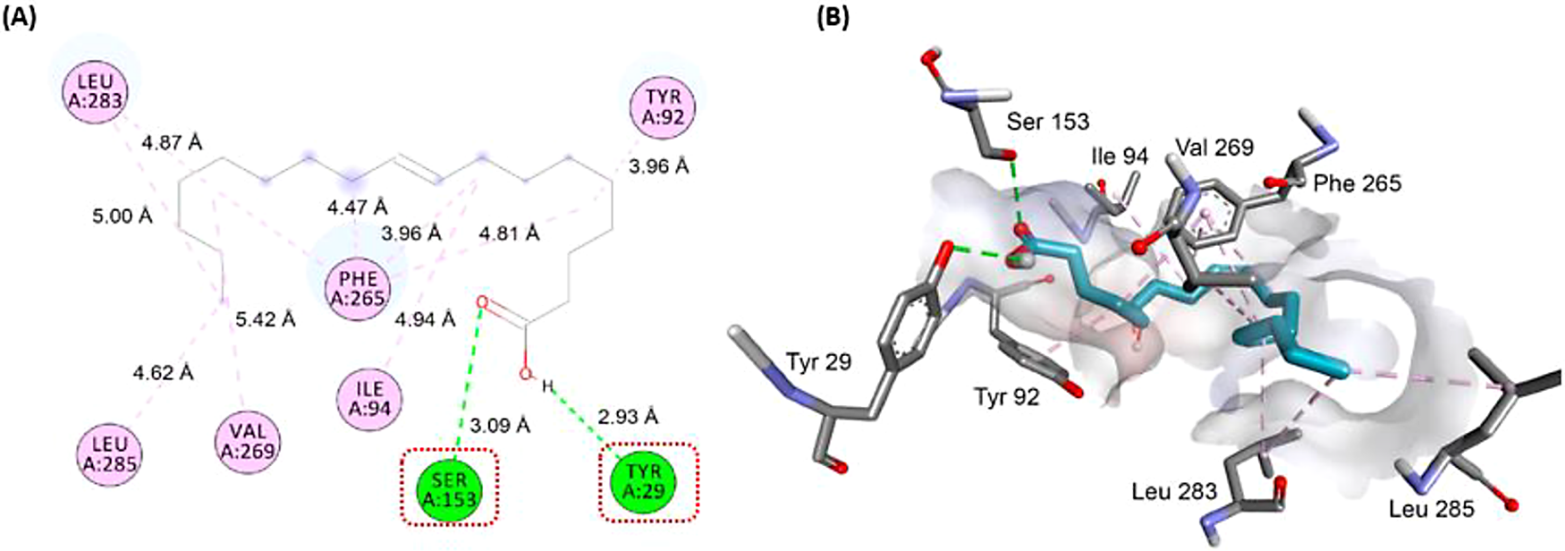
(A) Substrate in 2D: oleic acid, (B) oleic acid interactions between the catalytic triad of Eversa lipase Ser153-His268-Asp206 and amino acids residues.

The lower binding energy suggests that the combination of substrate with lipase is more stable and sufficient for esterification. Simulation results are shown in 2D in Figures 5 and 6. According to the molecular docking study, only the structures of the oleic acid compound interacted with the catalytic triad, more precisely with the carboxylic acid region close to serine 153. This interaction slightly favors the formation of an ester in the esterification reaction, according to the literature.^74^ This suggests that the triad remains active after immobilization for the bioreaction of Eversa with oleic acid. Linoleic acid also interacted with the enzyme residues with a binding affinity of −5.8 kcal/mol. Two hydrogen bonds were observed with Ser 153 and Tyr 29, critical regions for the esterification reaction. In addition, hydrophobic interactions were observed at Tyr 92, Ile 94, Phe 265, Val 269, Leu 283, and Leu 285, all of which were of the alkyl and π-alkyl type, as shown in Figure 6.

## Conclusions

Chemical modification of MNPs with APTES and glutaraldehyde has been validated as a potent strategy to improve the operational stability of immobilized enzymes. This modification confers resistance to oxidative forces that can degrade the integrity of the nanoparticles and strengthen the support against aggregation. Aldehyde functionalities from glutaraldehyde facilitate the formation of stable covalent bonds with amino groups on the enzyme, ensuring secure and durable attachment. This chemical synergy provides a favorable environment that preserves enzyme activity and prevents denaturation over successive catalytic cycles. By empirical observation, the ET 2.0-NPM biocatalyst showed exceptional efficiency in the synthesis of ethyl oleate, achieving a conversion rate of 88.1% under optimized conditions. This high conversion rate indicates the biocatalyst’s performance, suggesting that the enzyme’s structural integrity and catalytic potential are well preserved on the MNP support. The study also shows that the ET 2.0-NPM biocatalyst maintains significant operational stability over multiple reaction cycles. This stability is critical for industrial applications where the recovery and reuse of the biocatalyst can significantly reduce process costs and environmental impact. The ease of separation via a magnetic field further enhances the biocatalyst’s appeal as a sustainable alternative to conventional chemical catalysts, which often pose ecological hazards because of toxicity and disposal issues. Our results strongly suggest that using APTES and glutaraldehyde-modified MNPs is a superior approach for immobilizing enzymes used in biodiesel production. This technology can move the industry toward greener processes by providing a reusable, environmentally benign, and economically advantageous catalyst. In addition, magnetic recovery of the biocatalyst represents a significant advancement in addressing waste reduction and process optimization in biotechnology applications. Using silico analysis, oleic acid was found to bind near the enzyme’s active site with a binding free energy of −5.8 kcal/mol through hydrogen bonding, alkyl and π-alkyl interactions. For the Eversa homology model, the Ramachandran plot showed 91.5% of its residues in the favorable regions, 6.5% in the additional allowed regions, 1.6% in the generously allowed regions, and 0.4% in the unfavorable areas, indicating a good structure.

## Supporting Information Available

Figure S1: two-dimensional structure of oleic acid; Figure S2: proton ^1^H NMR spectrum of ethyl oleate, showing the main chemical shift signals and their multiplicities, obtained at a frequency of 500 MHz in deuterated solvent; Figure S3: chromatogram of the diluted ethyl oleate sample using a C-18 reversed-phase chromatographic column; Table S1: oil compositions’ respective percentage values for the identified molecules; and Table S2: results of molecular docking of oleic acid with the Eversa enzyme (PDF)
