# Supplementary material for: Enhanced Biodiesel Production with Eversa Transform 2.0 Lipase on Magnetic Nanoparticles
Source: Langmuir. 2024 Nov 26;40(51):26835–51. doi: 10.1021/acs.langmuir.4c02542 (PMC11673575; doi:10.1021/acs.langmuir.4c02542)
Supplement: Supplementary file 1 — la4c02542_si_001.pdf [file la4c02542_si_001.pdf]

SUPPORTING INFORMATION

Enhanced Biodiesel Production with Eversa® Transform 2.0 Lipase on Magnetic Nanoparticles

Kaiany Moreira dos Santos[1], Juliana de França Serpa[1], Viviane de Castro Bizerra[1], Rafael Leandro Fernandes Melo[2], Paulo Gonçalves de Sousa Junior[1], Valdilane Santos Alexandre[1], Aluísio Marques da Fonseca[1], Pierre Basílio Almeida Fechine[3], Diego Lomonaco[4], José Cleiton Sousa dos Santos[1,*], Maria Cristiane Martins de Souza [1]

[1]Instituto de Engenharia e Desenvolvimento Sustentável - IEDS, Campus das Auroras, Universidade da Integração Internacional da Lusofonia Afro-Brasileira - UNILAB, Rua José Franco de Oliveira, s/n - Zona Rural, Redenção 62790-970, CE, Brazil; moreirakaiany@gmail.com; serpajuli@hotmail.com; vivianebizerra15@gmail.com; paulogdsj@gmail.com; alexandrevaldilane@gmail.com; aluisiomf@unilab.edu.br; jcs@unilab.edu.br; mariacristiane@unilab.edu.br

[2]Departamento de Engenharia Metalúrgica e de Materiais, Universidade Federal do Ceará – UFC, Campus do Pici, Bloco 729, Fortaleza CEP 60440-554, CE, Brazil; rafael.melo@ifce.edu.br

[3]Departamento de Química Analítica e Físico-Química, Universidade Federal do Ceará - UFC, Campus do Pici, Bloco 940, Av. Humberto Monte, 2825, CEP 60455760, Fortaleza, CE, Brazil; fechine@ufc.br

[4]Laboratório de Produtos e Tecnologia em Processos (LPT), Universidade Federal do Ceará – UFC, Fortaleza 60440-900, CE, Brasil; lomonaco@ufc.br

***Corresponding author:**

Prof. Dr. José Cleiton Sousa dos Santos; e-mail: jcs@unilab.edu.br (J.C.S.S.)

Institute of Engineering and Sustainable Development University of International Integration of Afro-Brazilian Lusophony Redenção, CE, Brazil, Zip-Code: 62790970

The provided supporting information includes:

Figure S1: Two-dimensional structure of oleic acid.

Figure S2: ^{1}H NMR spectrum of ethyl oleate showing the main chemical shift signals and multiplicities obtained at a frequency of 500 MHz in deuterated solvent.

Figure S3: Chromatogram of the diluted sample for ethyl oleate on a C-18 reverse phase chromatographic column.

Table S1: Oil composition with their respective percentage values for the identified molecules.

Table S2: Oleic acid molecular docking results with eversa enzyme.

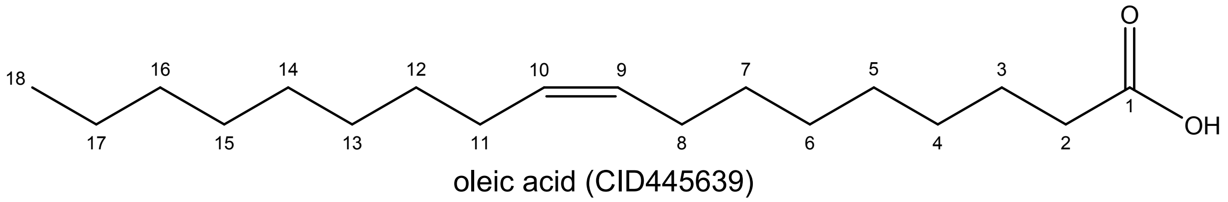

Figure S1. Two-dimensional structure of oleic acid.

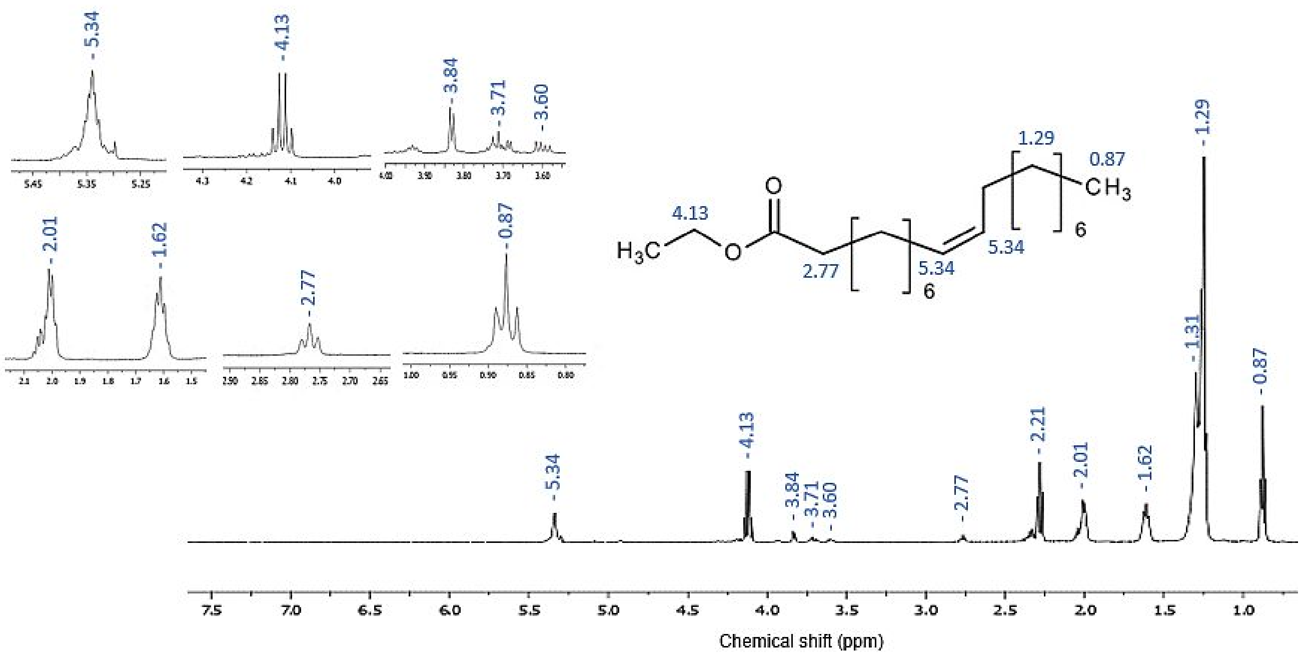

Figure S2. ^{1}H NMR spectrum of ethyl oleate showing the main chemical shift signals and multiplicities obtained at a frequency of 500 MHz in deuterated solvent.

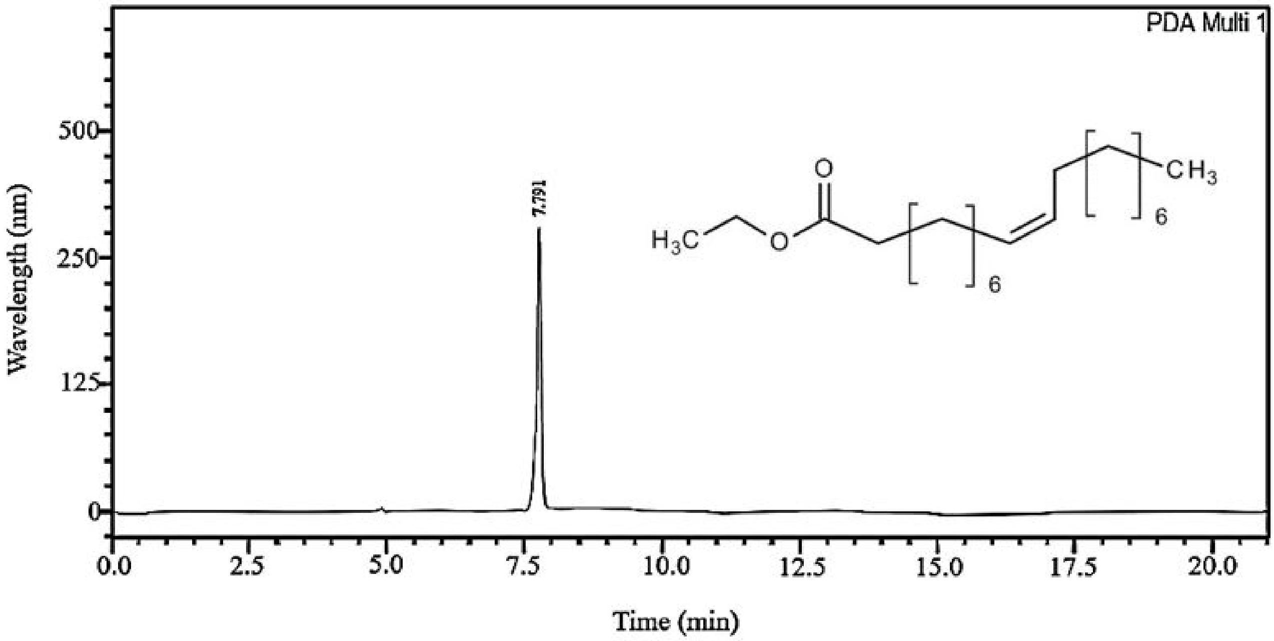

Figure S3. Chromatogram of the diluted sample for ethyl oleate on a C-18 reverse phase chromatographic column.

Table S1. Oil composition with their respective percentage values for the identified molecules.

**ESTER CONTENT (C) = 89%**	
**(%)**	**Molecule**
0,8%	Tetradecanoic acid
0,5%	Decanedioic acid, diethyl ester
1,6%	Ethyl tridecanoate
7,3%	n-Hexadecanoic acid
9,4%	Hexadecanoic acid, ethyl ester
80,3%	oleic acid methyl ester
100%	-

Table S2: Oleic acid molecular docking results with eversa enzyme.

Compounds	Energy (kcal/mol)	RMSD (Å)
Oleic acid (CID445639)	-5.8	2.00
